# What makes public health studies ethical? Dissolving the boundary between research and practice

**DOI:** 10.1186/1472-6939-15-61

**Published:** 2014-08-08

**Authors:** Donald J Willison, Nancy Ondrusek, Angus Dawson, Claudia Emerson, Lorraine E Ferris, Raphael Saginur, Heather Sampson, Ross Upshur

**Affiliations:** 1 University of Toronto, Dalla Lana School of Public Health, 155 College Street, 6th Floor, Toronto, ON M5T 3 M7, Canada; 2 Institute of Health Policy, Management and Evaluation, University of Toronto, 155 College Street, Suite 425, Toronto, ON M5T 3 M6, Canada; 3 Department of Clinical Epidemiology and Biostatistics, McMaster University, 1280 Main St W, Hamilton, ON L8S 4 K1, Canada; 4 Public Health Ontario, Suite 300, 480 University Avenue, Toronto, ON M5G 1 V2, Canada; 5 Medicine, Ethics, Society and History (MESH), 90 Vincent Drive, School of Health and Population Studies, College of Medical and Dental Sciences, University of Birmingham, Edgbaston, Birmingham, West Midlands B15 2TT, UK; 6 Sandra Rotman Centre – University Health Network & University of Toronto, MaRS Centre, South Tower, 101 College St., Suite 406, Toronto, ON M5G 1 L7, Canada; 7 Department of Philosophy, McMaster University, 1280 Main St W, Hamilton, ON L8S 4 K1, Canada; 8 Ottawa Hospital Research Institute, 725 Parkdale Ave, Ottawa, ON K1Y 4E9, Canada; 9 Bridgepoint Health, 14 St Matthews Rd, Toronto, ON M4M 2B5, Canada; 10 Department of Family and Community Medicine, University of Toronto, 500 University Avenue, 5th Floor, Toronto, ON M5G 1 V7, Canada; 11 Toronto East General Hospital, 825 Coxwell Avenue, Toronto, ON M4C 3E7, Canada

**Keywords:** Ethics, Public health, Research, Quality improvement, Program evaluation

## Abstract

**Background:**

The generation of evidence is integral to the work of public health and health service providers. Traditionally, ethics has been addressed differently in research projects, compared with other forms of evidence generation, such as quality improvement, program evaluation, and surveillance, with review of non-research activities falling outside the purview of the research ethics board. However, the boundaries between research and these other evaluative activities are not distinct. Efforts to delineate a boundary – whether on grounds of primary purpose, temporality, underlying legal authority, departure from usual practice, or direct benefits to participants – have been unsatisfactory.

Public Health Ontario has eschewed this distinction between research and other evaluative activities, choosing to adopt a common framework and process to guide ethical reflection on all public health evaluative projects throughout their lifecycle – from initial planning through to knowledge exchange.

**Discussion:**

The Public Health Ontario framework was developed by a working group of public health and ethics professionals and scholars, in consultation with individuals representing a wide range of public health roles. The first part of the framework interprets the existing Canadian research ethics policy statement (commonly known as the TCPS 2) through a public health lens. The second part consists of ten questions that guide the investigator in the application of the core ethical principles to public health initiatives.

The framework is intended for use by those designing and executing public health evaluations, as well as those charged with ethics review of projects. The goal is to move toward a culture of ethical integrity among investigators, reviewers and decision-makers, rather than mere compliance with rules. The framework is consonant with the perspective of the learning organization and is generalizable to other public health organizations, to health services organizations, and beyond.

**Summary:**

Public Health Ontario has developed an ethics framework that is applicable to any evidence-generating activity, regardless of whether it is labelled research. While developed in a public health context, it is readily adaptable to other health services organizations and beyond.

## Background

The practice of public health involves both the generation and use of a wide variety of evidence for decision-making. Evaluation routinely accompanies the introduction of new public health programs and services or the modification of established programs. Surveillance programs monitor indicators of health status as well as social and environmental conditions that may affect public health. Quality improvement activities collect data to ensure that ongoing programs and services are being implemented to appropriate standards. Research serves to address broader knowledge gaps to inform all of these activities.

Traditionally, ethics has been addressed differently in research projects, as compared with quality improvement, program evaluation, and surveillance, with only research requiring formal review by a research ethics board (REB). However, all these initiatives involve the systematic collection of data about individuals, their communities, their environments, and the health and social services they receive. They require either the primary collection of new data or the use of existing data for these purposes. They frequently use common methods and pose common risks to participants. Ethical issues may arise at any point in the conduct of any of these activities. Otto and colleagues point out that distinguishing between research and public health practice is particularly challenging, as public health practice may include hypothesis testing and use of the same epidemiologic study design, sampling and analysis techniques as a research project [1]. While not necessarily intended, public health practice may also lead to generalizable knowledge and be published [1].

There is general agreement that some form of ethical reflection or review is important for all types of evidence-generating initiatives, whether or not they constitute formal research [2-6]. It is widely suggested that review of non-research activities falls outside the purview of the REB [2,5,7,8]. However, the establishment of separate review processes for research and other types of evidence-generating initiatives is problematic because it requires one to differentiate between research and these other non-research initiatives. Efforts to delineate such a boundary [3,4,9,10] – whether on grounds of primary purpose (generalizable knowledge), temporality (turn-around time from evidence to action), underlying legal authority, departure from usual practice, or direct benefits to participants – have been unsatisfactory.

The wisdom of maintaining this artificial division between research and other evaluative activities has been debated in a number of circles in recent years. Fairchild and Bayer [11] note:

The recent efforts to provide definitional solutions to the question of research and public health practice involve twists and turns that inevitably produce results that are riddled with inconsistencies and that are conceptually unsatisfying.

In the context of clinical care and research, Kass and colleagues have systematically delineated the conceptual, moral, and empirical problems with attempting to draw a sharp distinction between clinical research and practice, with a view to taking a more holistic approach [12].

In summary, there is a growing recognition that the distinction between research and other evidence-generating activities is artificial and selective ethics review of activities labeled “research” is problematic. With this in mind, we sought to develop a common ethics framework for all evidence-generating activities, regardless of the label.

## Discussion

### The context

Public Health Ontario (PHO) is a government agency charged with the protection and promotion of health and the reduction of health inequities for Ontarians [13]. Through legislation, it operates as a separate entity from the government but within a broader accountability framework set in government directives and procedures. The services offered by PHO are broad in nature and include: research, program evaluation, surveillance and population health assessment. As a new agency, PHO had no prior policies or infrastructure to address the ethical review of research or other evaluative activities conducted by its staff. Recognizing the challenges with the blur between research and other evaluative activities, we sought to adopt a holistic approach to the ethical conduct of these initiatives. We found no literature describing any other organization that had done this. So, with the support of our President and CEO, we set out to develop a conceptual framework for the ethical conduct of public health initiatives that addresses, in an integrated fashion, research, surveillance, and other evidence-generating activities involving humans, their data or their biological samples, proportionate to the risk to human participants [14]. The use of a single system avoids the problems associated with trying to distinguish research from non-research described above, and ensures that all initiatives receive ethics assessment proportionate to the risk.

The PHO framework was developed by a working group of public health and ethics professionals and scholars, in consultation with individuals representing a wide range of public health roles. The concepts in the document were presented at various fora and early drafts were circulated to public health practitioners and ethicists for comment. The resulting feedback was incorporated into the first completed version of the framework document, which was released broadly for discussion in June 2011 to public health units across Ontario, independent academics working in the areas of public health and ethics, and individuals at specific organizations described in the acknowledgements. These comments informed the revision of the framework document into its current version.

### Why build on a research ethics paradigm?

The framework consists of two parts. The first part interprets the existing Canadian research ethics policy statement through a public health lens. The second part consists of ten questions that guide the investigator in the application of the core ethical principles to public health initiatives.

Our framework builds on the joint research ethics policy statement of the three federal research agencies in Canada, commonly known as the TCPS 2 [8]. All Canadian institutions must agree to comply with the TCPS 2 to be eligible to administer funds from the three federal research agencies in Canada. Like many other research guidance documents, TCPS 2 has been criticised for being too strongly driven by the biomedical research paradigm. We considered developing or adapting an ethics framework that was specifically grounded in public health values. The decision to use the TCPS 2 and its three core principles of respect for persons, concern for welfare, and justice as the starting point was based on a number of considerations:

– We sought to create a single process for all our public health evaluative activities while remaining compliant with TCPS 2 requirements for research.

– The foundational principles of TCPS 2 – respect for persons, concern for welfare and justice – are consonant with other internationally accepted bioethics principles. While additional principles that are particularly relevant to public health may also be introduced, the three principles of the Policy serve as the core.

– Concerns about the limitations of the TCPS 2 largely relate to misapplication. Perceived challenges with the applicability of the TCPS 2 in many instances reflect overly rigid interpretations amidst a cultural bias towards individual autonomy and a focus on avoiding risk, and not limitations in the actual guidance provided by the TCPS 2.

### Ten guiding questions

The public health lens we apply to the TCPS 2 considers how the three core principles may be read in the context of community or population interests, expanding the ethical considerations to include: the relational interests of the individual as part of a community (relational autonomy); solidarity; social justice; and reciprocity. To facilitate application of the three principles to specific projects, the PHO framework poses ten guiding questions to be considered when planning and reviewing evidence-generating public health initiatives. In Table 1, we provide a brief synopsis of the public health framing of each of the ten questions. The full discussion may be found elsewhere [14] It is important that the guiding questions be considered together and that they not be used in isolation from the discussion regarding their interpretation and application through a public health lens.

**Table 1:** Ten guiding questions

**No.**	**Question**
**1**	**What are the objectives of the initiative? How are they linked to potential improvements in public health?**
	− A clear link must be provided between the initiative and potential public health improvements; potential benefits may be immediate or future. Collection of data where public health value is more speculative may be permissible with justification.
	− This question serves as an anchor for review, as many of the questions below relate back to the original objectives.
**2**	**Can the objectives be achieved using the proposed methods?**
	− Initiatives lacking sufficient methodological rigour may lead to data that is of poor quality or invalid, wasting resources and potentially causing potential harm through misinformation.
	− Requirements for scientific rigour must be balanced with sensitivity to the context in which an activity is implemented.
	− Judgment regarding the design of an initiative requires relevant methodological expertise as well as some knowledge about the participating populations and other contextual details, as relevant.
**3**	**Who are the expected beneficiaries of the knowledge gained or other benefits?**
	− Beneficiaries may include individuals and/or communities, whether or not they are directly participating in the proposed initiative.
	− Individual and collective interests may be shared or competing, or both, depending on the circumstance.
**4**	**What are the burdens and potential harms associated with the proposed initiative? Who bears them?**
	− Harms associated with evidence generation in public health frequently arise from collection, use or disclosure of information; potential consequences include stigmatization, discrimination, psychological distress or economic loss. Other harms, such as threats to health, may also occur.
	− Burdens generally are borne by those participating in an initiative. Harms may affect individuals and/or communities, whether or not they are directly participating in the proposed initiative.
	− Potential harm to relationships should be considered.
	− Where possible, an effort must be made to mitigate or minimize risks and burdens, balancing against any loss in potential benefit.
**5**	**Are burdens and potential harms justified in light of the potential benefits to participants and/or to society?**
	− Burdens and potential harms should be weighed against not only potential benefit from conducting an inquiry, but the harm in not carrying out that inquiry.
	− Burdens or harms may accrue to different individuals/groups than those receiving the benefit but, where this is the case, there should be some justification.
	− “Fair procedures” such as transparency and stakeholder participation should be used to guide decision making regarding balancing of burdens, harms and benefits.
**6**	**Is selection of participants fair and appropriate?**
	− Fair distribution of burdens, risks and potential benefits includes paying special attention to vulnerable or disadvantaged populations, to be included where there is potential benefit, excluded where certain groups face greater burden or risk, or preferentially
	− Included because of increased probability or magnitude of benefit.
	− The principle of reciprocity requires finding ways to give back to individuals or communities that bear a disproportionate share of burden or risk for the benefit of others.
**7**	**Is individual informed consent warranted? Is it feasible? Is it appropriate? Is it sufficient?**
	− While important, individual autonomy does not always take priority over other ethical concerns, such as welfare of populations.
	− For many public health initiatives, obtaining individual consent may not be required, feasible or appropriate. Where departure from individual informed consent is proposed, consider alternatives such as broad consent, notice with opt out, and consultation with a representative sample of the population of interest.
	− In certain cases, such as examination of illegal behaviour, alternative approaches such as use of verbal consent or pseudonyms may be appropriate.
**8**	**Is community engagement warranted? Is it feasible? What level of engagement is appropriate?**
	− Community engagement is encouraged where feasible and might be used in lieu of, or in addition to individual consent.
	− Engagement may range from informing to consultation, collaboration and empowerment.
	− Community engagement may include some form of collective consent or consensus process authorizing the initiative in the community.
	− Challenges include determining what level of engagement is appropriate, what counts as a community, and who the appropriate representatives are.
**9**	**What are the social justice implications of this initiative?**
	− Projects that reinforce existing inequities should be avoided and opportunities to promote social justice should be considered where possible.
	− Extra resources or special measures may be needed to promote social justice, for example to ensure that disadvantaged groups are appropriately considered in the development of project objectives, or to remove barriers to their participation in public health initiatives.
**10**	**What are the potential longer-term consequences?**
	− Where possible, potential negative long-term consequences of an initiative should be considered and plans for mitigating these risks should be developed prior to implementation.
	− Community engagement can be helpful both in identifying potential long term harms, and in devising methods to address them.

These questions are informed by several earlier frameworks developed for clinical research [15], public health [16], and health services research [2]. Many of the questions themselves do not differ from what REBs typically address when reviewing a research protocol. The added value we provide is the public health perspective in the explanatory text – e.g. considering harm to communities, reciprocity, and the levelling of autonomy with other principles.

Question ten may be the most contentious. It asks about the potential longer-term consequences, with an emphasis on unanticipated adverse effects of a study. Some research guidance specifically identifies this question as being out of scope of ethics review [17]. While this may be reasonable in an academic setting, in the applied world of public health one must consider the potential implications of an evaluation on future policy.

### Application of the PHO framework

The PHO framework is more than a tool for the external review of a public health protocol. It was designed for use by those who are developing the protocol – from the formulation of objectives through to reporting of the findings [18]. We believe this will assist public health professionals in taking ownership of the ethical issues inherent in their practice, rather than complying with requirements imposed by an external ethics authority. This is particularly important in emergency situations, where action may need to be taken before formal ethics review could take place.

We recognize that extending ethics review to all knowledge-generating activities has major workload implications for an institution’s ethics oversight process. To address this issue, we are rolling out a number of tools and processes to manage the increased volume of projects that will come under scrutiny. These include:

a) a risk screening tool that creates a new risk category for minimal risk evaluative activities that have relatively well defined risks and which can be reviewed by the research ethics officer for appropriate measures to mitigate those risks.

b) the development of standardized protocols for certain types of routine evaluative activities that are pre-reviewed for ethics.

Once evaluated, these will be the subjects of future papers.

Adoption of this framework by public health organizations will require training of ethics review board members to manage the cultural shift to this framework from the traditional model of ethics review. This shift may not be particularly challenging, given the alignment of the framework with widely accepted public health principles. At PHO, we have used the framework as the basis for reviewer assessment forms, so that the ten questions are systematically applied to each project.

### The PHO Framework in the context of other calls for ethics reform

Our PHO framework is consonant with a recent call for an ethics framework for the learning health care system, which also advocates for the integration of rather than the distinction between research and practice [19]. That framework is structured around the obligations of multiple parties to contribute to the common purpose of improving the quality and value of clinical care and health care systems. Our framework particularly resonates with the last three obligations: to reduce health inequalities among populations; to conduct responsible activities that foster learning from clinical care and clinical information; and to contribute to the common purpose of improving the quality and value of clinical care and health care systems. With regard to the last obligation, the authors identify an obligation of the individual patient to contribute, under limited conditions, to learning that is integrated with their care. Similarly, public health is founded on the assumption that there are certain common goods that ought to be promoted. Rather than referring to obligations of individual patients, we have framed this in terms of the inter-dependence among members within and between communities and draw upon the principle of solidarity to promote our collective welfare.

While we acknowledge that the current research oversight system is often too bureaucratic, we do not agree with calls for exemption from ethics review *as a class* for surveys, focus groups, and similar research involving competent adults, as has been advocated in some circles [20]. There may still be non-trivial risks associated with these data collection activities – particularly in public health, where one may be working with vulnerable populations, asking sensitive questions, or working with information that is not in the public domain. Consequently, at a minimum, all new evaluative studies involving human participants must complete the risk screening tool and undergo an initial screen by the ethics officer.

We are unaware of any published literature describing institutions that have taken an integrated perspective similar to that which we have put forward. Anecdotally, as we developed our framework, we learned that several local public health units also review quality improvement and research studies through the same process, but they had not created a comprehensive conceptual framework like ours. This may well be the case with other institutions.

## Summary

Public Health Ontario has developed a framework to guide ethical reflection on all public health evaluative projects throughout their lifecycle – from initial planning through to knowledge exchange – eschewing the distinction between research and other evaluative activities. The framework is intended for use by those designing and executing public health evaluations, and not only for those charged with ethics review of projects.

While we have built upon the TCPS 2’s three core principles of respect for persons, concern for welfare, and justice, we have augmented these with concepts of: relational autonomy and respect for communities; the inter-relationship of individual and community welfare; solidarity and the common good; the positive obligation to promote social justice; and the importance of reciprocity in circumstances when individuals or subgroups put themselves at risk or bear substantial burden for the benefit of others.

The use of guiding questions rather than statements of principles or rules was chosen to help move toward a culture of ethical integrity among investigators, reviewers and decision-makers, rather than one of compliance with rules. We believe this to be consistent with the perspective of the learning organization. We believe the framework is generalizable to public health practitioners in other jurisdictions and, with appropriate adaptation, even beyond public health to any discipline engaged in applied evaluation of public programs and services including health services, education, and social sciences.

## Competing interests

The authors declare that they have no competing interests.

## Authors’ contributions

DW conceptualized and led the development of the ethics framework. He oversaw the writing and revision of the framework report and wrote the first draft of this manuscript, along with subsequent revisions. NO was coordinator for this project and was responsible for writing and revising the drafts of the full framework report, published on the Public Health Ontario website. She was also integrally involved with reviewing and revising this manuscript. AD, CE, LF, RS, HS, and RU were all members of the working group that developed the ethics framework. They all contributed substantively to review and revision of both the full framework document and this manuscript. All authors read and approved the final manuscript.

## Pre-publication history

The pre-publication history for this paper can be accessed here:

http://www.biomedcentral.com/1472-6939/15/61/prepub
